# Annotations

**Published:** 1910-03-05

**Authors:** 


### Annotations.

### The Congress of Physiotherapy.

The third International Congress and Exhibition of Physiotherapy will be held in Paris from March 29 to April 3 this year, under the presidency of Professor Landouzy, the doyen of the Parisian Faculty of Medicine. The President of the French Republic has consented to become the patron of the Congress, which will be held on the premises of the faculty, the practical school and the well-known "Street of the School of Medicine" being covered over for the occasion with waterpoof canvas so as to form a vast exhibition hall. The importance of physiotherapy is now well recognised, and the Congress has done good work in the past, and will do much excellent service in the future, by drawing the attention of the profession to new advances and discoveries in the domain of physiotherapy. We are informed that a strong French Committee has been formed, which has extended a cordial invitation to English practitioners to attend the Congress and visit the exhibition, which bids fair to be one of the most interesting professional shows organised in recent years. A very attractive programme of lectures, entertainments, visits, and demonstrations has been drawn up, and the English and French railway companies have consented to issue special return tickets at reduced fares to intending members of the Congress and their families. It is worth while to point out that the Congress meets during the Easter vacation, when the French capital is at its best and gayest—an added inducement for those who wish to combine pleasure with profitable instruction! The London agent of the Congress is M. Saunion, of H. Saunion and Cie, 19 Bartholomew Close, E.C., from whom all details regarding the arrangements may be obtained.

### The Tropical Research Fund.

The Blue Book (Cd. 4999) report of the Advisory Committee for the Tropical Diseases Research Fund was issued last week, and contains much interesting details regarding the advances in the treatment and prophylaxis of tropical disorders. The income of the Fund, from various sources, was £3,470, of which £3,333 was distributed in grants to the London and Liverpool Schools and the Universities of London and Cambridge, to pay for additional lecturers and demonstrators, or expenses in connection with special research. The Committee has suffered a severe loss through the death of Sir Ralph Moor, whose active interest in its work was of the greatest value in past years. The report records the successful establishment and organisation of the Australian Institute of Tropical Medicine, towards the equipment of which a grant of £400 was made from the Fund in 1908. Among other developments of the year has been the establishment of a small bacteriological laboratory at St. Lucia, and the appointment of a bacteriologist to that island. The Sleeping Sickness Bureau has done excellent service during the short time of its existence, and promises to become of important and invaluable assistance to those interested in this branch of tropical medicine. Finally, the report records the praiseworthy energy of the administrations of the various colonies in supporting the Fund and co-operation in the work of prevention and eradication of tropical disease that is now being carried on.

### The Anæsthetics Bill and the General Medical Council.

At the Executive Committee's meeting of the General Medical Council on February 21 last, a communication was considered from the Privy Council drawing attention to the recommendation of the Royal College of Surgeons with reference to the fifth clause in the draft Anæsthetics Bill. This clause reads as follows: "That, having regard to existing conditions, it is also desirable in the public interest that duly qualified dental practitioners should be authorised to administer certain specified anæsthetics, such as nitrous oxide gas, for the purpose of inducing unconsciousness or insensibility to pain during dental operations or procedures." The Council of the R.C.S. recommends that the words "during dental operations or procedures" should be omitted on the ground that, "having regard to the fact that the services of a medical practitioner with special experience in the administration of anæsthetics are often not available . . . it is not to the advantage of the public or of the medical profession that dentists should be debarred from administering gas and the other anæsthetics mentioned in the schedule for operations performed by duly qualified medical practitioners." This appears to us to be a sensible conclusion, but the General Medical Council has not been able to see it in that light. After discussion it was resolved: "That the Lord President be informed that the words proposed to be omitted were inserted advisedly by the Council, and in the opinion of the Executive Committee it is desirable in the public interest that they should be retained. The Executive Committee would point out that cases may occur in which, during the course of medical or surgical operations, commenced under anæsthesia produced by nitrous oxide or other 'specified' preparation, it is found necessary at a later stage to resort to more potent anæsthetics such as chloroform or ether. In such a case it might endanger the patient's safety, and the success of the operation, were the anæsthetist unqualified to administer the latter, as under the legislation proposed an anæsthetist possessed only of a dental qualification would be." We do not think that this is a conclusion with which most practitioners or the profession as a whole will agree. Certainly in private practice—and here only would the dentist's aid be invoked—no such contingency as the Council contemplates is likely to arise. No practitioner will undertake operations in which anæsthesia produced by nitrous oxide gas has to be continued or followed by chloroform or ether. We cannot recall a single instance in private practice where such a course as the General Medical Council has cited was followed, and in the circumstances we still believe that the recommendation of the Royal College is sound and ought to be followed.

